# Arithmetic Errors in Financial Contexts in Parkinson’s Disease

**DOI:** 10.3389/fpsyg.2021.629984

**Published:** 2021-04-14

**Authors:** Hannah D. Loenneker, Sara Becker, Susanne Nussbaum, Hans-Christoph Nuerk, Inga Liepelt-Scarfone

**Affiliations:** ^1^Department of Psychology, Diagnostics and Cognitive Neuropsychology, Eberhard Karls Universität Tübingen, Tübingen, Germany; ^2^Department of Neurodegeneration, Hertie Institute for Clinical Brain Research, Tübingen, Germany; ^3^German Centre for Neurodegenerative Diseases (DZNE), Tübingen, Germany; ^4^IB Hochschule für Gesundheit und Soziales, Stuttgart, Germany

**Keywords:** dyscalculia, financial management, neurodegeneration, MCI, elderly, gender differences, attention, visuo-spatial function

## Abstract

Research on dyscalculia in neurodegenerative diseases is still scarce, despite high impact on patients’ independence and activities of daily living function. Most studies address Alzheimer’s Disease; however, patients with Parkinson’s Disease (PD) also have a higher risk for cognitive impairment while the relation to arithmetic deficits in financial contexts has rarely been studied. Therefore, the current exploratory study investigates deficits in two simple arithmetic tasks in financial contexts administered within the Clinical Dementia Rating in a sample of 100 PD patients. Patients were classified as cognitively normal (PD-NC) or mildly impaired (PD-MCI) according to Level I consensus criteria, and assessed using a comprehensive neuropsychological test battery, neurological motor examination, and sociodemographic and clinical questionnaires. In total, 18% showed arithmetic deficits: they were predominately female, had longer disease duration, more impaired global cognition, but minor signs of depression compared to PD patients without arithmetic deficits. When correcting for clinical and sociodemographic confounders, greater impairments in attention and visuo-spatial/constructional domains predicted occurrence of arithmetic deficits. The type of deficit did not seem to be arbitrary but seemed to involve impaired place × value processing frequently. Our results argue for the importance of further systematic investigations of arithmetic deficits in PD with sensitive tests to confirm the results of our exploratory study that a specific subgroup of PD patients present themselves with dyscalculia.

## Introduction

Arithmetic function deteriorates with age (Stemmler et al., 2013) and underlies elderly independent living skills (i.e., financial management; Finke et al., 2017). Despite this importance for activities of daily living (ADL), research on arithmetic deficits in elderly is scarce and primarily conducted in children (Kaufmann et al., 2013; Knops et al., 2017). Within the elderly population, neurodegeneration increases susceptibility to arithmetic deficits (e.g., Kalbe, 1999; Halpern et al., 2003; Arcara et al., 2019). While most research has been conducted in Alzheimer’s Disease (AD), there are first hints that patients with other dementias, such as Parkinson’s Disease (PD) dementia (PDD), also present with dyscalculia (Kalbe, 1999). Even though one might expect arithmetic deficits to have been assessed thoroughly given the extensive profiling of cognitive impairment in PD, research is scarce (e.g., Kalbe, 1999; Tamura et al., 2003). Unsystematic clinical observations show arithmetic errors in both advanced PDD (Kalbe, 1999) and non-demented PD patients (Tamura et al., 2003; Zamarian et al., 2006; Scarpina et al., 2017).

Previous research in PD operationalized arithmetic uniformly. However, developmental studies show dyscalculia resulting from distinct impairments in both specific numerical and domain-general cognitive functions (Jordan and Montani, 1997; Kaufmann et al., 2013). Domain-general cognitive functions required for arithmetic arise from different neuropsychological domains such as attention, working memory, language, executive or visuo-spatial function (Knops et al., 2017). Specific numerical prerequisites for arithmetic are heterogeneous, with magnitude being a core representation (e.g., Dehaene and Cohen, 1995). Furthermore, the application of calculation procedures is essential and impaired in AD (Mantovan et al., 1999). Another basis of multi-digit arithmetic is place-value integration (i.e., identification, activation, manipulation of digits within and between Arabic numbers; Nuerk et al., 2015). Analyzing specific errors then allows to infer underlying mechanisms (Nuerk et al., 2015): Erroneous magnitude processing shows as rounding errors within the correct decade (i.e., 3 × 6 = 16 not 18). Impaired calculation procedures can arise as operand (i.e., 3 × 6 = 12) or operation errors (i.e., 3 × 6 solved as 3+6). Errors regarding decade value (i.e., 3 × 6 = 28), place (3 × 6 = 180) or both (3 × 6 = 280) stress an impaired place-value integration.

Financial capabilities are distinct and multidimensional skills (Marson, 2001, 2013), with arithmetic abilities such as number comprehension, principles, mental and written calculation being crucial prerequisites. Other cognitively mediated skills such as global cognitive function, short-term and working memory, (verbal) memory and learning, executive function, visuo-motor skills, decision making, financial conceptual knowledge, or instrumental ADL are associated with financial capabilities (Sherod et al., 2009; Lichtenberg et al., 2016; Arcara et al., 2019). Due to their complexity as higher order cognitive functions, financial capacities are prone to processes of aging and neurodegeneration (Willis, 1996; Marson et al., 2000). As arithmetic functioning is crucial, the current study explores PD-immanent arithmetic errors in financial contexts.

Arithmetic–specific cognitive deficits have not been well studied in PD yet possibly due to focusing on motor symptoms. Nowadays, PD is defined as a multisystem disorder affecting motor, autonomous, psychiatric and cognitive function (Postuma et al., 2015), with cognitive impairments often being confounded with motor symptoms (Das et al., 2016). PD-specific cognitive classifications continuously range from normal cognition (PD-NC) over mild cognitive impairment (PD-MCI) to PDD (Aarsland, 2016). These cognitive profiles are heterogeneous; old age, male gender, cortical cerebrospinal fluid (CSF) amyloid-beta 1–42 (Aβ42) pathology, depression and, most importantly presence of PD-MCI indicate susceptibility for PDD conversion (Irwin et al., 2012; Marras and Chaudhuri, 2016; Aarsland et al., 2017; Lin et al., 2018). Several factors, such as education, gender or work experience, have been shown to affect (numerical) healthy aging (Delazer et al., 2013; Lövdén et al., 2020) and might influence PD patients’ arithmetic ability. However, the relationship between these specific profiles and arithmetic deficits has not been studied in PD, indicating the need to characterize patients making arithmetic errors to diagnose them timely for early interventions (Tucker-Drob, 2019).

Furthermore drawing inferences how arithmetic deficits affect PD patients from previous research focusing on AD is difficult, as similarity in clinical profiles is limited despite neuropathological overlaps (e.g., cholinergic impairments; Bohnen et al., 2003). Arithmetic deficits in AD (Rosselli et al., 1998) and early impairment of complex financial capacity in prodromal AD or MCI (Triebel et al., 2009; Marson, 2013, 2015) suggest a possible diagnostic value of arithmetic function for cognitive deterioration in PD, requiring clarification. Both the cognitive stage where arithmetic deficits first occur and the quality of impairments remain unknown. Arithmetic function in financial contexts is important for the autonomy and legal responsibilities of PD patients (Sherod et al., 2009; Marson, 2013; Arcara et al., 2019). Therefore, alteration in number cognition might also be arise in a prodromal stage of PDD, being investigated in this study. Therefore, it is crucial to phenotype arithmetic deficits by defining cognitive profiles of affected patients which can be achieved by addressing associations to other cognitive functions as suggested by different PD stages showing distinct cognitive profiles (Lopes et al., 2017).

The aim of the current study is (H1) to identify the frequency of arithmetic errors in financial contexts in PD-NC and PD-MCI patients and (H2) to profile characteristic patients committing these errors regarding sociodemographic, clinical, and cognitive measurements as compared to arithmetically unaffected patients. The last hypothesis addresses (H3) whether errors PD patients make can be attributed to specific categories of numerical processing to infer affected cognitive mechanisms. These hypotheses were investigated with data available from a longitudinal study focusing on the predictive value of CSF Aβ42 pathology in PD at the University Hospital in Tübingen. It includes sociodemographic and clinical assessments, as well as a neuropsychological test battery. Two arithmetic tasks in financial contexts administered within the Clinical Dementia Rating (Morris, 1993) were used to identify whether financial-arithmetic capabilities are a question relevant for PD research.

## Materials and Methods

### Participants

Present data come from the longitudinal “Non-demented patients with Parkinson’s disease with and without low Amyloid-beta 1–42 in cerebrospinal fluid” (ABC-PD longitudinal) study, focusing on the predictive value of Aβ42 pathology for cognitive worsening. The study was approved by the local ethics committee (686/2013BO1). Participants were recruited via the outpatient PD clinic or the ward at Tübingen University Hospital’s neurology department. Patients received monetary compensation for travel expenses, and were assessed in the “on-state” with regular dopaminergic medication.

100 non-demented PD patients were selected by pre-screening neurological function confirming PD diagnosis following United Kingdom Brain-Bank criteria (Hughes et al., 1992). All patients received a lumbar puncture at least six weeks before the baseline visit and were between 50 and 85 years old. Patients were able to communicate well with the investigator, understand study requirements and give written informed consent. Diagnosis of PDD according to the Movement Disorder Society (MDS) Task Force criteria (Emre et al., 2007), other concomitant neurodegenerative diseases as well as substance abuse (except nicotine) led to participant exclusion.

Patients’ CSF Aβ42 status was determined using commercially available ELISA kits (INNOTEST; Fujirebio Germany GmbH, Hannover, Germany, RRID: AB_2797385). Patients were divided into two equal sized (*n* = 50) groups: Aβ42+ (<600 pg/mL) and Aβ42– (≥600 pg/mL). Groups were matched according to age, gender and educational status. For the present analysis, PD-MCI was diagnosed according to the Level I MDS Task Force criteria (Litvan et al., 2012). All 100 patients were included in the analyses.

### Material

#### Sociodemographic and Clinical Information

Demographics (age, gender, education years, disease duration, and age at PD onset) were acquired in an interview.

#### Arithmetic Function in Financial Contexts

Two standardized financial arithmetic tasks from the *Clinical Dementia Rating* interview (CDR; Morris, 1993; RRID:SCR_003678) were presented orally: *“How many 5 cent coins make up 1€?”* and *“How many 50 cent coins make up 15.50€?*” Answers in a verbal open response format were assessed based on correctness and errors where possible. Errors were ascribed to distinct categories (see Table 1 for details and examples): Place-value integration errors (i.e., wrong decade value, wrong place, both), magnitude related errors (i.e., rounding in correct decade), procedural errors (i.e., operand error, wrong operation), or other errors (i.e., arbitrary errors, operands’ repetition). Errors lacking information on participants’ exact answers were categorized as NA.

**TABLE 1:** Description of error categories with examples of patient answers.

		Patient answers	
Error category	Description	How many 5 cent coins make up 1€? = *20*	How many 50 cent coins make up 15.50€? = *31*
Place-value	Wrong decade value Wrong place Wrong place and decade value	10 2 or 200 100	11
Magnitude	Rounding within correct decade		30 or 32
Procedural	Operand error (solution in multiplication table) Wrong operation (division not multiplication) Wrong operation and place−value integration	15	7.75 76 (≈15.50 ÷ 2 ×10)
Others	Arbitrary Repetition of operand	4	4 or 8 or 23 or 25 or 26 15
NA	No information on exact error given		

#### Cognitive Function

The *Montreal Cognitive Assessment* (MoCA*;* Nasreddine et al., 2005) screened for global cognitive impairment (max. sum score 30 = normal cognitive performance). A MoCA score ≤ 26 indicated impaired global cognitive performance and assigned patients to the PD-MCI group. Cognitive function was additionally assessed with the German version of the *Repeatable Battery for the Assessment of Neuropsychological Status* (RBANS; Randolph et al., 1998). Scores on the twelve subtests were categorized into the domains attention, immediate and delayed memory, language, and visuo-spatial/constructional function (see Supplementary Material B for mapping of subtests to domains). Raw scores converted to age-group corrected z-scores, and composite domain and total scale scores according to the manual. The current analysis comprised RBANS domain and total scores.

#### Clinical Measurements

Parkinson’s Disease motor symptoms were evaluated using the sum score of the MDS Unified Parkinson’s Disease Rating Scale Part III (UPDRS III; Goetz et al., 2008) and Hoehn and Yahr staging (Hoehn and Yahr, 1967). The *UPDRS-III* rated motor symptoms on a scale ranging from 0 = normal to 4 = severe, with a maximum score of 132. The Hoehn and Yahr score ranging from one to four (1 = unilateral involvement; 4 = severe disability) additionally measured PD severity. Motor type was calculated from the UPDRS-III and item 12 from the former UPDRS-II version (Fahn et al., 1987) by means of the mean tremor score (postural, kinetic, or rest tremor) and the mean postural instability and gait disorder score (PIGD; falls, postural instability, freezing of gait). Patients were categorized as tremor-dominant in case of a ratio mean tremor score / mean PIGD score of 1.50 or higher or as PIGD dominant for ratios of 1.00 or lower, or as mixed for the remaining cases (Jankovic and Kapadia, 2001).

Anti-parkinsonian drug intake was expressed as *levodopa equivalent daily dose* (LEDD; Tomlinson et al., 2010). Patients’ *depressive symptoms* during the last two weeks were rated with the Beck Depression Inventory (BDI-II; Hautzinger et al., 2006). *Health-related quality of life* was assessed with the single index score of the 39-item Parkinson’s Disease Questionnaire (PDQ-39; Jenkinson et al., 1997). Items scored on a scale from 0 = never to 3 = often, with a successive transformation into weighted sum scores. The Functional Activities Questionnaire (FAQ; Pfeffer et al., 1982) was used to measure *activities of daily living function*. Patients rated their level of performance (0 = normal to 3 = dependent) on 10 ADLs subsumed as sum score.

### Procedure

Testing took place in Tübingen University Hospital. Patients gave written informed consent and were assessed for eligibility based on the in- and exclusion criteria. Additionally to study assessments, most patients had appointments in the Parkinson’s disease outpatient clinic before or after the study visit. Therefore, the order of clinical assessment varied between patients.

### Data Analysis

Assessments were analyzed manually and data was managed using REDCap electronic data capture tools (Harris et al., 2009; RRID: SCR_003445). Statistical analyses were conducted with R version 4.0.3 (R Core Team, 2014; RRID: SCR_001905) and JASP version 0.13.1 (JASP Team, 2018; RRID: SCR_015823). Due to the small patient samples, Gaussian distribution of data was not assumed resulting in analyses using median, range, Mann-Whitney *U* tests, χ^2^-tests, Brunner-Munzel tests (non-parametric trend test with ${\hat{p}}^{\prime \prime }$ = test statistic of stochastic equality; Brunner and Munzel, 2000), and binary logistic regressions. Confounders for the logistic regressions were chosen based on significantly differing variables between groups with and without arithmetic errors in financial contexts. Multicollinearity between predictors of the regression models was assessed based on a variance inflation factor (VIF) criterion above 10, not met by any predictor. For inferential statistics, an α-level of 0.05 was applied.

Analyses were conducted with the entire sample. Due to the over-representation of Aβ42+ patients, analyses were repeated with a subsample (*N* = 63) including all Aβ42- patients (*n* = 50) and a proportion of 20% Aβ42+ patients (*n* = 13). This reflects the estimated empirical distribution with a prevalence of AD pathology (Aβ42+, Tau, phosphorylated Tau) in approximately 30 to 40% of predominantly demented PD patients with non-demented PD patients falling considerably below this rate (Boller et al., 1980; Blennow and Hampel, 2003; Braak et al., 2005; Siderowf et al., 2010; Irwin et al., 2012). Unless indicated otherwise, outcomes did not differ between overall and representative sample (see Supplementary Material).

## Results

### Frequency of Arithmetic Errors in Financial Contexts in PD (H1)

The total sample included 42% PD-MCI patients. Overall, 18% of PD patients (PD-NC and PD-MCI) showed arithmetic errors in at least one of the two financial items. PD-MCI patients showed 1 or 2 errors more frequently (26.2%) than PD-NC patients (12.1%), however, this difference did not reach significance, ${\hat{p}}^{\prime \prime }$ = 1.74, *p* = 0.09. For the Aβ42 groups, 16.0% of positive and 20.0% of negative patients showed arithmetic errors; this was not statistically significant χ^2^(1) = 0.27, *p* = 0.60.

The amount of errors differed marginally significantly between PD-NC (0 errors: 87.9%, 1 error: 8.6%, 2 errors: 3.4%) and PD-MCI (0 errors: 73.8%, 1 error: 4.8%, 2 errors: 21.4%), ${\hat{p}}^{\prime \prime }$ = −1.92, *p* = 0.059. The binary logistic regression correcting for the influence of gender, disease duration, and depression [*χ^2^*(94) = 11.92, *p* = 0.018, $R_{\text{McFadden}}^{2}$ = 0.088, Area under the curve (AUC) = 0.695] revealed the amount of errors was the only significant predictor of cognitive status, with PD-MCI displaying more errors than PD-NC (*p* = 0.004). This model did not reach significance with the reduced representative sample (see Supplementary Material A). For analyses of RBANS subtests see Supplementary Material B (gender, depression, story memory predict arithmetic errors; differences between arithmetic groups: digit span, list learning, list recognition, picture naming, semantic fluency, line orientation).

### Phenotyping Arithmetic Errors in PD (H2)

Patients with arithmetic errors differed from those without regarding gender (more females), disease duration (longer), depression (lower BDI-II scores), and global cognition (lower MoCA total and RBANS total scale scores, see Table 2). On average, females (0 error: 63.6%, 1 error: 12.1%, 2 errors: 24.2%) committed more errors than males (0 error: 91%, 1 error: 4.5%, 2 errors: 4.5%), ${\hat{p}}^{\prime \prime }$ = 3.01, *p* = 0.004. Effects were the same in the representative sample, except for groups not differing statistically regarding disease duration and depression (Supplementary Material, Table A1).

**TABLE 2:** Sociodemographic and clinical characterization of study patients.

	Total sample *N* = 100	Min. 1 arithmetic error *n* = 18	No arithmetic error *n* = 82	*p*
Age	65.49 (50.52–80.36)	66.80 (54.21–79.58)	64.60 (50.52–80.36)	0.15
Male *n* (%)	67.00 (67.00%)	6.00 (33.30%)	61.00 (74.40%)	<0.001*
Education years	13.00 (8.00–21.00)	12.00 (9.00–18.00)	13.00 (8.00–21.00)	0.16
Disease duration	4.17 (0.81–14.57)	6.51 (1.34–14.57)	3.98 (0.81–13.10)	0.02*
Age at onset	59.50 (38.94–77.53)	59.30 (39.79–74.78)	59.80 (38.94–77.53)	0.71
Aβ42+ status *n* (%)	50.00 (50.00%)	8.00 (44.40%)	42.00 (51.90%)	0.60
Motor type *n* (%)				0.87
PIGD	46.00 (46.00%)	9.00 (50.00%)	37.00 (45.10%)	
Mixed	10.00 (10.00%)	2.00 (11.10%)	8.00 (9.80%)	
Tremor dominant	44.00 (44.00%)	7.00 (38.90%)	37.00 (45.10%)	
UPDRS-III	25.00 (5.00–56.00)	23.00 (10.00–54.00)	25.00 (5.00–56.00)	0.89
Hoehn and Yahr score *n* (%)				0.25
1	3.00 (3.00%)	1.00 (5.60%)	2.00 (2.40%)	
2	79.00 (79.00%)	12.00 (66.70%)	67.00 (81.70%)	
3	18.00 (18.00%)	5.00 (27.80%)	13.00 (15.90%)	
LEDD	560 (100–2077.25)	627.50 (250–1353)	546.00 (100–2077.25)	0.42
BDI-II	6.00 (0–28.00)	3.50 (0–12.00)	6.00 (0–28.00)	0.03*
PDQ-39 summary index	2.15 (0.08–17.92)	2.76 (0.08–10.74)	2.13 (0.10–17.92)	0.66
FAQ	0 (0–20.00)	0 (0–15.00)	0 (0–20.00)	1.00
MoCA total score	26.00 (14.00–30.00)	24.50 (14.00–29.00)	26.00 (17.00–30.00)	0.02*
RBANS total scale score	90.00 (54.00–127.00)	82.00 (54.00–106.00)	92.50 (54.00–127.00)	0.002*

Group comparisons were conducted with Mann-Whitney U tests or χ^2^ where appropriate; median (range) or frequencies are given as measures of central tendency. *p < 0.05. Arithmetic errors are defined as: one or both arithmetic tasks not solved correctly. UPDRS-III = Unified Parkinson’s Disease Rating Scale Part 3; LEDD = Levodopa equivalent daily dose; BDI = Beck Depression Inventory; PDQ-39 = Parkinson’s Disease Questionnaire 39; MoCA = Montreal Cognitive Assessment; RBANS = Repeatable Battery for the Assessment of Neuropsychological Status. Due to missing values, the overall sample was reduced to N = 99 for BDI-II, PDQ-39, and FAQ.

Results of the binary logistic regression indicated a significant association of gender, disease duration, depression, and all RBANS domain scores with the presence of arithmetic errors, *χ^2^*(90) = 51.12, *p* < 0.001, $R_{\text{McFadden}}^{2}$ = 0.545, AUC = 0.941. Female gender, long disease duration, low depression scores, impaired attention and visuo-spatial/constructional deficits significantly predicted arithmetic errors (see Table 3). In the representative sample, only attention was a significant predictor for study group (see Supplementary Material, Table A2). In a second binary logistic regression model, confounding variables (gender, disease duration, depression) influenced presence of arithmetic errors *χ^2^*(94) = 22.00, *p* < 0.001, $R_{\text{McFadden}}^{2}$ = 0.234, AUC = 0.822, while the FAQ score did not (see Table 3). This model was not stable in the representative sample (see Supplementary Material A).

**TABLE 3:** Results of the binary logistic regressions predicting arithmetic errors in financial contexts.

						Wald Test			95%-CI		
	*B*	SE	*β*	OR	z	Wald statistic	df	*p*	Lower bound	Upper bound	VIF
**1) Model including RBANS cognitive domain scores and covariates**											
Intercept	16.58	6.23	–3.81	15860000	2.66	7.08	1	0.008*	4.37	26.79	
Gender	–3.29	1.11	–1.56	0.04	–2.97	8.79	1	0.003*	–5.47	–1.12	1.90
Disease duration	0.36	0.14	1.31	1.44	2.54	6.44	1	0.011*	0.08	0.64	1.51
BDI-II	–0.37	0.12	–2.31	0.69	–2.98	8.87	1	0.003*	–0.61	–0.13	1.92
Attention	–0.10	0.05	–1.72	0.91	–2.09	4.36	1	0.037*	–0.19	–0.01	3.12
Immediate memory	0.03	0.04	0.56	1.03	0.83	0.68	1	0.410	–0.04	0.10	2.88
Delayed memory	0.002	0.03	0.03	1.00	0.07	0.01	1	0.945	–0.06	0.07	1.99
Language	–0.034	0.05	–0.35	0.97	–0.63	0.40	1	0.529	–0.14	0.07	2.17
Visuo-spatial/constructional	–0.10	0.04	–1.35	0.91	–2.31	5.33	1	0.021*	–0.18	–0.01	1.94
**2) Model including ADL function and covariates**											
Intercept	–0.94	0.68	–2.07	0.39	–1.38	1.91	1	0.167	–2.26	0.39	
Gender	–1.72	0.63	–0.82	0.18	–2.76	7.59	1	0.006*	–2.95	–0.50	1.07
Disease duration	0.19	0.08	0.69	1.21	2.28	5.20	1	0.023*	0.03	0.36	1.14
BDI-II	–0.15	0.07	–0.96	0.86	–2.32	5.36	1	0.021*	–0.28	–0.02	1.23
FAQ	0.07	0.08	0.29	1.08	0.93	0.87	1	0.350	–0.08	0.28	1.31

*p < 0.05. B = estimated regression coefficient; SE = Standard error; β = standardized regression coefficient; OR = Odds Ratio; df = degrees of freedom; CI = confidence interval of the estimate; BDI = Beck Depression Inventory; FAQ = Functional activity questionnaire. Arithmetic errors level ‘1’ was coded as class “min. 1 error” and gender level ‘1’ was coded as “male.”

### Categorization of Errors (H3)

Arithmetic tasks differed regarding error categories in the entire patient sample. For the 5 cent task, place-value integration errors were most frequent (54.5%), followed by procedural (9.1%) and other errors (9.1%). In the 50 cent task, most errors could not be categorized (33.3%) or were magnitude-related (33.3%), followed by procedural (11.1%) and place-value integration errors (5.6%). Importantly, 27.3% (5 cent) and 16.7% (50 cent) of cases were NAs. The proportion of error categories did not differ between cognitive groups or by gender, but descriptively, more place-value integration errors occurred in the 5 cent task compared to the 50 cent task (see Table 4).

**TABLE 4:** Proportion of error categories in relation to total errors as percentages per cognitive status and gender.

Error category	How many 5 cent coins make up 1€?						How many 50 cent coins make up 15.50€?					
	Cognitive status		*p*	Gender		*p*	Cognitive status		*p*	Gender		*p*
	PD-MCI	PD-NC		Male	Female		PD-MCI	PD-NC		Male	Female	
Place-value	55.6%	50.0%	1.00	66.7%	50.0%	1.00	9.1%	0%	1.00	16.7%	0%	0.25
Magnitude	0%	0%		0%	0%		27.3%	42.9%		16.7%	41.7%	
Procedural	11.1%	0%		0%	12.5%		9.1%	14.3%		0%	16.7%	
Others	11.1%	0%		33.3%	0%		45.5%	14.3%		50.0%	25.0%	
NA	22.2%	50.0%		0%	37.5%		9.1%	28.6%		16.7%	16.7%	

*Group comparisons were conducted with χ^2^ tests and frequencies are given as measures of central tendency. Both NAs (wrong answers without specification of the error committed) and the category others were excluded from the χ^2^ tests.*

## Discussion

The current study aimed to identify the frequency of financial-arithmetic impairments in PD subgroups, as well as in relation to sociodemographic, clinical, and cognitive factors. Results demonstrate clinically relevant arithmetic errors in financial contexts. Identified risk factors were female gender, longer disease duration, greater severity of depressive symptoms, and more cognitive impairment. Place-value integration- and magnitude-related errors were most frequent. Cognitive groups differed regarding the amount of errors in some analyses. Note the limitation that arithmetic errors increased for longer and cognitively more severe PD, but patients were not compared with controls.

### Frequency of Arithmetic Errors in Financial Contexts in PD (H1)

The overall frequency of arithmetic errors of 18% in two simple tasks supports the need for further systematic investigation. When correcting for clinical confounders, the amount of errors was able to correctly predict cognitive status where higher errors were indicative of PD-MCI. However, some PD-NC patients also showed arithmetic errors, similar to previous AD studies finding dyscalculia in early stages (Parlato et al., 1992; Martin et al., 2003). Therefore, PD patients showing heterogeneous arithmetic impairments even in early stages and its association to specific cognitive profiles demands further examination. The model using the representative sample did not reach significance, which needs to be interpreted with care due to: a smaller sample (63 instead of 100 patients), an associated decrease in arithmetic errors, a greater tendency to ceiling effects, and a smaller amount of explained variance. Based on the current findings, it is impossible to infer on global financial capacities, as these are defined multidimensionally and exceed arithmetic function alone. However, showing a difference in arithmetic errors between PD-NC and PD-MCI indicates an association of PD disease severity and the likelihood of arithmetic errors in financial contexts.

### Phenotyping Arithmetic Errors in Financial Contexts in PD (H2)

Profiles of PD patients with arithmetic errors were female, longer disease duration, less depression, and more cognitively impaired. Total MoCA and RBANS scale scores differed significantly between arithmetic groups, suggesting an association between arithmetic errors and cognition in PD. As PD-MCI and longer disease duration predict PDD (Aarsland et al., 2017), our data suggest that arithmetic errors occur within the frame of heterogeneous progressive cognitive deterioration.

When analyzing the proportion of errors (0,1,2), a systematic effect of gender (women made more errors) and cognitive status (PD-MCI patients made more errors) was observed, even after correcting for confounders. However, when error frequency was aggregated (1 or 2 errors in one category), when confounders were not considered, or when a smaller sample was used with a proportion of Aβ42+, differences between PD-NC and PD-MCI were only trends. We attribute this lack of significance when error categories are grouped to a statistical power issue due to sample sizes, tendency toward ceiling effects in the more representative sample, or decreased explained variance. While these results suggest more severe arithmetic errors in more cognitively impaired PD patients, they also advise for a future more systematic assessment.

The binary logistic regressions showed attention, visuo-spatial/constructional function, and story memory predicted arithmetic errors, suggesting degeneration in these domains at least partly causing arithmetic deficits. Current research in PD also discusses the importance of attention and visuo-spatial function for cognitive status und progression to PDD, introducing these domains as candidates for early biomarkers (Lopes et al., 2017; Becker et al., 2020). Associations between visuo-spatial/constructional functions and numerosity in healthy adults are in line with finding visuo-spatial/constructional functions to predict arithmetic errors in the current study (Lammertyn et al., 2002; Thompson et al., 2013). Furthermore, retrieving arithmetic facts requires an intact verbal memory (Dehaene and Cohen, 1997). Interestingly, females showed worse arithmetic performance than males. The gender differences in favor of men are in line with findings by Delazer et al. (2013) and Arcara et al. (2019). They explain advantages of elderly men in arithmetic and financial capabilities with employment in mathematics-related fields, higher level of education (paralleling generational effects) and mathematical interests. The arithmetic advantage for men is remarkable as gender effects usually dissociate from men showing stronger global cognitive decline but indicate differentiated visuo-spatial and verbal memory deficits in female PD patients (Fengler et al., 2016; Bakeberg et al., 2021). Therefore, the current association of visuo-spatial/constructional functions and story memory with arithmetic deficits is in line with finding more arithmetic deficits in female patients.

We also found that patients with arithmetic errors were, on average, less depressed than those without. This contradicts research on numeracy skills negatively affecting mental health in elderly (Fastame et al., 2019), and depression impairing cognitive performance in PD (Alzahrani and Venneri, 2015). As the difference in arithmetic does not seem to be PD-specific or directly related to cognition, the association with gender and education should be addressed to differentiate a coincidental finding from a systematic effect of depression on arithmetic in PD.

The binary logistic regression including ADL explained no more than a small amount of variance in arithmetic errors. The insignificant effect might be explained by patients in the current study being less heavily impacted on ADL, with ADL impairment occurring later in the process of transition from PD-MCI to PDD (Becker et al., 2020).

### Presence of PD-Specific Error Categories (H3)

Most observed errors were place-value integration errors, followed by magnitude-related or procedural errors. Therefore, our data provide first evidence that not all numerical representations are impaired alike in PD. Future research should examine the validity of these results in a systematic methodological setup, with multi-digit and complex arithmetic tasks, enough items for a broader investigation of errors, and enough statistical power to identify PD-specific error categories and differences between cognitive statuses.

### Limitations and Future Studies

The current exploratory study indicates the presence of particular arithmetic error types in financial contexts in PD, which – in some analyses – seem to associate with cognitive decline. These findings are novel in a hitherto neglected research field. However, this study has a couple of limitations, requiring consideration in follow-up studies.

First, the comparison with a healthy elderly group is missing to estimate the extent of impairments. However, finding arithmetic errors in both groups of PD-NC and PD-MCI with arithmetic errors to increase alongside progression of cognitive status indicates differing arithmetic impairments in discrete PD stages. Future studies should include healthy controls for comparisons between PD and the general population as well as more cognitively nuanced PD groups. Second, the methodological approach is not sufficient to provide generalizable inferences on arithmetic errors in financial contexts in PD with a rate of 18%. Yet, these errors are not negligible, but are absent in the diagnostic criteria for cognitive deficits in PD. Third, there were only two items and one type of arithmetic problem. Future studies should employ more systematic and broader assessments (e.g., different operations, different magnitudes, different place-value processing procedures, verbal and non-verbal tasks, symbolic and non-symbolic tasks) to obtain a more comprehensive overview on which errors are PD-specific and how pronounced they are for different numerical representations and processes. Fourth, this study is cross-sectional; however, longitudinal studies, informative for characterizing neurodegenerative processes when appropriately correcting for practice effects and selective attrition (Moody et al., 2017; Tucker-Drob, 2019), are missing. These designs can identify person-to-person heterogeneity in trajectories of lifespan cognitive developments being specific for the respective cognitive ability and interdependent for individuals (see Tucker-Drob, 2019). Heterogeneous arithmetic deficits found in AD (Girelli and Delazer, 2001) stress the need for differential investigations in PD.

### Conclusion

In conclusion, while the current study reports first interesting data about arithmetic errors in financial contexts in PD, it is a mere starting point and inspiration to investigate such errors more systematically in the future. The current study suggests: (1) PD patients show arithmetic errors in financial contexts which seem to be more pronounced with cognitive impairment, (2) error type does not seem to be arbitrary but hints at a predominantly impaired place-value processing, and (3) apart from PD-MCI status there is heterogeneity within the groups and distinct attributes such as attention, visuo-spatial/constructional function, gender, or disease duration influence the likelihood of arithmetic errors, (4) The male advantage in arithmetic processing in PD contrasts men’s larger global cognitive decline but follows female visuo-spatial and story memory disadvantages. In sum, we believe that these results suggest arithmetic performance in financial contexts to be a problem in PD-MCI, deserving future attention.

## Data Availability Statement

The datasets presented in this article are not readily available because public accessibility to the data has not been included in the ethics approval and participant’s informed consent. Requests to access the datasets should be directed to HL, hannah-dorothea.loenneker@uni-tuebingen.de.

## Ethics Statement

The studies involving human participants were reviewed and approved by Ethik-Kommission an der Medizinischen Fakultät der Eberhard-Karls-Universität und am Universitätsklinikum Tübingen (686/2013BO1). The patients/participants provided their written informed consent to participate in this study.

## Author Contributions

IL-S and H-CN performed conceptualization. SB and SN performed data curation and investigation. HL performed formal analysis, visualization, and writing original draft. IL-S and HL performed funding acquisition. IL-S, HL, and H-CN performed methodology. SB, SN, and IL-S performed project administration. IL-S performed resources, supervision, and validation. HL, SB, H-CN, and IL-S performed writing review and editing the manuscript. All authors contributed to the article and approved the submitted version.

## Conflict of Interest

This study received funding from Janssen Research and Development, a division of Janssen Pharmaceutica N.V. The study is purely precompetitive. The funder was not involved in the study design and data collection. Additionally, the funder was not involved in the current analysis, interpretation of data, the writing of this article, or the decision to submit it for publication. The authors declare that the research was conducted in the absence of any commercial or financial relationships that could be construed as a potential conflict of interest.
