# Supplementary material for: Arithmetic Errors in Financial Contexts in Parkinson’s Disease
Source: Front Psychol. 2021 Apr 14;12:629984. doi: 10.3389/fpsyg.2021.629984 (PMC8079777; doi:10.3389/fpsyg.2021.629984)
Supplement: Supplementary file 1 [file Data_Sheet_1.docx]

Supplementary Material A: Analyses conducted with the representative sample regarding Aβ42 characteristics

# Frequency of arithmetic errors in financial contexts in Parkinson’s Disease (H1)

The representative sample (*n* = 63) included 44.4% Parkinson’s Disease (PD) patients with mild cognitive impairments (PD-MCI). Overall, 20.6% of PD patients showed arithmetic errors, operationalized as incorrect answers in at least one of the two arithmetic items. PD-MCI patients displayed arithmetic errors more frequently (28.6%) than PD-NC patients (14.3%), however this difference did not reach significance, $\hat{p}''$ = 1.35, *p* = .18.

Regarding the amount of errors committed, PD patients with normal cognition (PD-NC; 0 error: 85.7%, 1 error: 8.6%, 2 errors: 5.7%) and PD-MCI (0 error: 71.4%, 1 error: 3.6%, 2 errors: 25.0%) patients were not stochastically unequal $\hat{p}''$ = -1.52, *p* = .14. When correcting for the influence of the confounders gender, and education years (see Table 2) with a binary logistic regression on cognitive status, *χ^2^*(59) = 4.48, *p =* .214, $R_{McFadden}^{2}$.= .052, Area under the curve (AUC) = .628, the model did not reach significance. For groups split according to Aβ42 status, 23.1% of positive and 20.0% of negative patients showed arithmetic errors, χ^2^(1) = 0.06, *p* = .81.

# Phenotyping arithmetic errors in PD (H2)

The subgroups of patients with and without arithmetic errors differed regarding gender (more females showed errors), education years (errors occurred with less education), and global cognition (both MoCA and RBANS total scale score were lower for the patient group with arithmetic errors, see Table 1). Regarding the amount of errors committed, male (0 error: 88.6%, 1 error: 4.5%, 2 errors: 6.8%) and female (0 error: 57.9%, 1 error: 10.5%, 2 errors: 31.6%) patients were stochastically unequal $\hat{p}''$ = 2.47, *p* = .02.

Table A1.

*Sociodemographic and clinical characterization of study patients.*

|  | Total sample  *N* = 63 | Min. 1 arithmetic error  *n* = 13 | No arithmetic error  *n* = 50 | *p* |  |
| --- | --- | --- | --- | --- | --- |
| Age | 65.51 (51.80- 80.36) | 70.00 (54.21- 79.58) | 64.50 (51.80- 80.36) | .06 |  |
| Male *n* (%) | 44 (69.8%) | 5 (38.5%) | 39 (78.0%) | .01 | * |
| Education years | 13 (8-21) | 12.00 (9-18) | 13.00 (8-21) | .05 | * |
| Disease duration | 5.13 (0.81-14.34) | 6.71 (2.64- 14.34) | 4.28 (0.81- 13.10) | .09 |  |
| Age at onset | 59.49 (38.94-76.29) | 59.50 (39.79- 74.78) | 59.40 (38.94- 76.29) | .31 |  |
| Aβ42+ status *n* (%) | 13 (20.6%) | 3 (23.1%) | 10 (20.0%) | 1.00 |  |
| Motor type *n* (%)  PIGD  Mixed  Tremor dominant | 30 (47.6%)  4 (6.3%)  29 (46.0%) | 6 (46.2%)  0  7 (53.8%) | 24 (48.0%)  4 (8.0%)  22 (44.0%) | .71 |  |
| UPDRS-III | 26 (5-56) | 22.00 (13.00-54.00) | 28.00 (5-56) | .45 |  |
| Hoehn & Yahr score *n* (%)  1  2  3 | 2 (3.2%)  50 (79.4%)  11 (17.5%) | 1 (7.7%)  10 (76.9%)  2 (15.4%) | 1 (2.0%)  40 (80.0%)  9 (18.0%) | .51 |  |
| LEDD | 580 (100-2077.25) | 640.00 (250- 1353) | 577.50 (100- 2077.25) | .83 |  |
| BDI-II | 6 (0-28) | 4.00 (0-12) | 6.00 (0-28) | .09 |  |
| PDQ-39 summary index | 2.47 (0.08-17.92) | 3.44 (0.08- 10.74) | 2.08 (0.10- 17.92) | .31 |  |
| FAQ | 1.0 (0-20) | 2.0 (0-15) | 0 (0-20) | .29 |  |
| MoCA total score | 26 (14-30) | 22.00 (14-29) | 26.00 (17-30) | .02 | * |
| RBANS total scale score | 89.00 (54-127) | 82.00 (54-103) | 90.5 (54-127) | .004 | * |

*Note.* Group comparisons were conducted with Mann-Whitney U tests or *χ^2^* as appropriate and median with range or frequencies are given as measures of central tendency. * = *p* < .05. Arithmetic errors are defined as: *one or both arithmetic tasks not solved correctly*. UPDRS-III = Unified Parkinson’s Disease Rating Scale Part 3; LEDD = Levodopa equivalent daily dose; BDI = Beck Depression Inventory; PDQ-39 = Parkinson’s Disease Questionnaire 39; MoCA = Montreal Cognitive Assessment; RBANS = Repeatable Battery for the Assessment of Neuropsychological Status. Due to missing values, the overall sample was reduced to *N* = 62 for BDI-II, PDQ-39, and FAQ.

Results of the binary logistic regression on arithmetic errors indicated that there was a significant association between gender, education years, and all RBANS domains, *χ^2^*(55) = 32.62, *p* < .001, $R_{McFadden}^{2}$.= .377, AUC = .860. None of the factors showed multicollinearity with all VIFs below ten, and attention was revealed to be the only significant predictor (see Table 2). Results of a second binary logistic regression indicated that there was no significant association between arithmetic errors and gender, education years, and activities of daily living, *χ^2^*(58) = 2.09, *p* = .555, $R_{McFadden}^{2}$.= .024, AUC = .709.

Table A2.

*Results of the binary logistic regression predicting arithmetic errors in financial contexts.*

|  |  |  |  |  |  | Wald Test | | | |  | 95%-CI | |  |
| --- | --- | --- | --- | --- | --- | --- | --- | --- | --- | --- | --- | --- | --- |
|  | *B* | SE | *β* | OR | z | Wald  statistic | df | *p* |  |  | Lower  bound | Upper  bound | VIF |
| Model including cognitive domains | | | | | | | | | | | | | |
| Intercept | 14.90 | 5.80 | -0.18 | 296800 | 2.57 | 6.61 | 1 | .010 | * |  | 3.54 | 26.26 |  |
| Gender | -1.00 | 0.96 | -0.46 | 0.37 | -1.04 | 1.07 | 1 | .300 |  |  | -2.89 | 0.89 | 1.67 |
| Education years | 0.23 | 0.18 | 0.63 | 1.26 | 1.32 | 1.74 | 1 | .188 |  |  | -0.11 | 0.58 | 1.43 |
| Attention | -0.06 | 0.03 | -1.13 | 0.94 | -2.11 | 4.46 | 1 | .035 | * |  | -0.11 | 0.00 | 2.06 |
| Immediate memory | -0.06 | 0.03 | -1.05 | 0.95 | -1.81 | 3.29 | 1 | .070 |  |  | -0.11 | 0.00 | 3.45 |
| Delayed memory | 0.02 | 0.03 | -0.35 | 0.98 | -0.69 | 0.48 | 1 | .490 |  |  | -0.08 | 0.04 | 2.24 |
| Language | -0.04 | 0.05 | -0.40 | 0.96 | -0.78 | 0.61 | 1 | .436 |  |  | -0.15 | 0.06 | 2.05 |
| Visuo-spatial | -0.01 | 0.03 | -0.11 | 0.99 | -0.25 | 0.07 | 1 | .800 |  |  | -0.07 | 0.05 | 1.81 |

*Note.* * = *p* < .05. *B* = estimated regression coefficient, SE = Standard error, *β* = standardized regression coefficient, OR = Odds Ratio, df = degrees of freedom, CI = confidence interval of the estimate. Arithmetic errors level '1' was coded as class "min. 1 error" and gender level '1' was coded as "male".

# Categorization of errors (H3)

When classifying errors, categories were represented differently in the two tasks. In the 5 cent task (*How many 5 cent coins make up 1€?*), place−value integration errors were committed most frequently (44.4%), followed by procedural (11.1%) and other errors (11.1%). In the 50 cent task (*How many 50 cent coins make up 15.50€?*), most errors were magnitude-related (38.5%) or could not be categorized (30.8%), followed by place−value integration errors (7.7%). It is important to state that the lack of information regarding errors committed (5 cent task: 33.3% NA, 50 cent task: 23.1% NA) is relatively large. Proportion of error categories did not differ by cognitive status or gender (see Table 3).

Table A3.

*Proportion of error categories in relation to total errors as percentages per cognitive status and gender.*

| Error category | How many 5 cent coins make up 1€? | | | | | | How many 50 cent coins make up 15.50€? | | | | | |
| --- | --- | --- | --- | --- | --- | --- | --- | --- | --- | --- | --- | --- |
|  | Cognitive status | | *p* | Gender | | *p* | Cognitive status | | *p* | Gender | | *p* |
|  | PD-MCI | PD-NC |  | Male | Female |  | PD-MCI | PD-NC |  | Male | Female |  |
| Place− value | 42.9% | 50.0% | 1.00 | 66.7% | 33.3% | 1.00 | 12.5% | 0% | 1.00 | 20.0% | 0% | .33 |
| Magnitude | 0% | 0% |  | 0% | 0% |  | 25.0% | 60.0% |  | 20.0% | 50.0% |  |
| Procedural | 14.3% | 0% |  | 0% | 16.7% |  | 0% | 0% |  | 0% | 0% |  |
| Others | 14.3% | 0% |  | 33.3% | 0% |  | 50.0% | 0% |  | 40.0% | 25.0% |  |
| NA | 28.6% | 50.0% |  | 0% | 50.0% |  | 12.5% | 40.0% |  | 20.0% | 25.0% |  |

*Note.* Group comparisons were conducted with *χ^2^*^-^-tests and frequencies are given as measures of central tendency. Both *NAs* (wrong answers without specification of the error committed) and the rest category *others* were excluded from the *χ^2^*^—^tests.

Supplementary Material B: Descriptive analysis of RBANS domain and subtest scores

Table B1.

*RBANS domain and subtest scores by arithmetic error and cognitive status groups.*

|  | Arithmetic errors | |  | Cognitive status | |  |
| --- | --- | --- | --- | --- | --- | --- |
|  | Min. 1 error  *n* = 18 | No error  *n* = 82 | *p* | PD-MCI  *n* = 42 | PD-NC  *n* = 58 | *p* |
| Attention | 82.00 (46.00-115.00) | 94.00 (56.00-135.00) | <.001 | 85.00 (46.00-118.00) | 100.00 (72.00-135.00) | <.001 |
| Digit span | -0.56 (-3.90-2.24) | -0.10 (-1.76-2.76) | .02 | -0.56 (-3.90-2.29) | 0.37 (-2.00-2.76) | <.001 |
| Coding | -2.10 (-3.56-1.65) | -0.68 (-4.44-2.10) | .002 | -1.50 (-4.44-0.75) | -0.20 (-3.03-2.10) | <.001 |
| Immediate memory | 95.50 (53.00 123.00) | 101.50 (57.00-129.00) | .10 | 88.50 (53.00-123.00) | 109.00 (73.00-129.00) | <.001 |
| List learning | -0.90 (-2.92-1.11) | 0.22 (-2.72-2.22) | .02 | -1.02 (-2.92-1.11) | 0.36 (-2.52-2.22) | <.001 |
| Story memory | -0.54 (-3.17-1.83) | 0.17 (-3.83-1.60) | .41 | -0.69 (-3.83-1.31) | 0.45 (-1.54-1.83) | <.001 |
| Delayed memory | 88.00 (52.00-112.00) | 98.50 (48.00-122.00) | .02 | 88.50 (48.00-119.00) | 101.00 (71.00-122.00) | <.001 |
| List recall | -0.62 (-2.73-2.04) | 0.44 (-2.73-2.04) | .15 | -0.42 (-2.73-2.04) | 0.45 (-1.96-2.04) | <.001 |
| List recognition | -0.33 (-4.33-0.67) | 0.50 (-2.83-0.86) | .05 | -0.25 (-4.33-0.67) | 0.50 (-2.83-0.86) | .006 |
| Story recall | -0.48 (-2.73-1.29) | -0.05 (-3.00-1.36) | .15 | -0.88 (-3.00-1.29) | 0.33 (-2.05-1.36) | <.001 |
| Figure recall | -1.31 (-2.65-0.85) | -0.45 (-3.40-1.36) | .004 | -0.90 (-3.40-1.36) | -0.40 (-2.26-1.07) | .03 |
| Language | 97.50 (82.00-112.00) | 96.00 (60.00-127.00) | .56 | 92.00 (60.00-113.00) | 103.00 (82.00-127.00) | <.001 |
| Picture naming | 0.58 (-1.40-0.67) | 0.60 (-3.71-0.90) | .10 | 0.60 (-3.71-0.90) | 0.60 (-0.86-0.90) | .03 |
| Semantic fluency | -0.25 (-2.61-1.19) | -0.40 (-3.42-2.39) | .42 | -0.92 (-3.42-1.19) | 0.23 (-1.80-2.39) | <.001 |
| Visuo-spatial/ constructional | 75.00 (60.00-100.00) | 87.00 (56.00-116.00) | .001 | 78.00 (56.00-112.00) | 87.00 (60.00-116.00) | .003 |
| Figure copy | -2.54 (-4.33-0.11) | -1.56 (-4.89-1.22) | .16 | -2.41 (-4.89-0.53) | -1.56 (-4.43-1.22) | .13 |
| Line orientaton | -0.86 (-4.79-0.83) | 0.21 (-4.79-1.29) | <.001 | -0.21 (-4.79-1.29) | 0.48 (-4.79-1.29) | <.001 |

*Note.* Descriptive statistics of central tendency and variance are displayed as median with range. Groups were compared with Mann-Whitney U tests. Interpretation of group comparisons needs to take multiple comparisons into account.

Results of a binary logistic regression on arithmetic deficits indicated that there was a significant association between gender, disease duration, depression, and all RBANS subtests, *χ^2^*(83) = 57.29, *p* < .001, $R_{McFadden}^{2}$.= .610, AUC = .961. When checking for multicollinearity, the variance inflation factor (VIF) exceeded the value of ten for the RBANS subtests list learning (VIF = 12.56) and list recall (VIF = 16.26). Gender, depression, and story memory were revealed to be significant predictors, whereas disease duration, digit span, coding, and line orientation showed additional statistical trends.

Table B2.

*Results of the binary logistic regression on RBANS subtests predicting arithmetic errors in financial contexts.*

|  |  |  |  |  |  | Wald Test | | | |  | 95%-CI | |  |
| --- | --- | --- | --- | --- | --- | --- | --- | --- | --- | --- | --- | --- | --- |
|  | *B* | SE | *β* | OR | z | Wald  statistic | df | p |  |  | Lower  bound | Upper  bound | VIF |
| Intercept | -2.31 | 1.48 | -4.96 | 0.10 | -1.56 | 2.44 | 1 | .118 |  |  | -5.20 | 0.59 |  |
| Gender | -4.43 | 1.60 | -2.10 | 0.01 | -2.78 | 7.72 | 1 | .005 | * |  | -7.56 | -1.31 | 3.54 |
| Disease duration | 0.36 | 0.19 | 1.30 | 1.44 | 1.91 | 3.66 | 1 | .056 | (*) |  | -0.01 | 0.73 | 1.93 |
| BDI-II | -0.48 | 0.19 | -3.03 | 0.62 | -2.49 | 6.20 | 1 | .013 | * |  | -0.87 | -0.10 | 4.43 |
| Digit span | -1.22 | 0.68 | -1.50 | 0.30 | -1.79 | 3.19 | 1 | .074 | (*) |  | -2.55 | 0.12 | 2.20 |
| Coding | -1.36 | 0.80 | -1.71 | 0.26 | -1.71 | 2.91 | 1 | .088 |  |  | -2.93 | 0.20 | 5.96 |
| List learning | -1.27 | 1.15 | -1.65 | 0.28 | -1.10 | 1.20 | 1 | .273 |  |  | -3.53 | 1.00 | 12.56 |
| Story memory | 1.83 | 0.87 | 2.25 | 6.25 | 2.10 | 4.40 | 1 | .036 | * |  | 0.12 | 3.54 | 6.53 |
| List recall | 1.76 | 1.17 | 2.19 | 5.80 | 1.51 | 2.27 | 1 | .132 |  |  | -0.53 | 4.04 | 16.26 |
| List recognition | 0.15 | 0.61 | 0.18 | 1.16 | 0.24 | 0.06 | 1 | .812 |  |  | -1.06 | 1.35 | 3.81 |
| Story recall | -1.70 | 1.08 | -1.84 | 0.18 | -1.58 | 2.49 | 1 | .115 |  |  | -3.81 | 0.41 | 7.25 |
| Figure recall | 0.01 | 0.78 | 0.01 | 1.01 | 0.02 | 0.00 | 1 | .986 |  |  | -1.52 | 1.55 | 3.46 |
| Picture naming | -0.06 | 0.89 | -0.03 | 0.94 | -0.07 | 0.00 | 1 | .948 |  |  | -1.81 | 1.69 | 1.80 |
| Semantic fluency | -0.71 | 0.76 | -0.77 | 0.49 | -0.92 | 0.85 | 1 | .356 |  |  | -2.20 | 0.79 | 4.84 |
| Figure copy | -0.17 | 0.37 | -0.25 | 0.85 | -0.44 | 0.20 | 1 | .658 |  |  | -0.89 | 0.56 | 1.65 |
| Line orientation | -0.75 | 0.41 | -0.90 | 0.47 | -1.84 | 3.38 | 1 | .066 | (*) |  | -1.55 | 0.05 | 2.08 |

*Note.* * = *p* < .05, (*) = statistical tendency. *B* = estimated regression coefficient, SE = Standard error, *β* = standardized regression coefficient, OR = Odds Ratio, df = degrees of freedom, CI = confidence interval of the estimate. Arithmetic errors level '1' was coded as class "min. 1 error" and gender level '1' was coded as "male".
